# Research: Treatment Factors associated with statin treatment for the primary prevention of cardiovascular disease in people within 2 years following diagnosis of diabetes in Scotland, 2006–2008

**DOI:** 10.1111/dme.12409

**Published:** 2014-03-24

**Authors:** N R V Jones, C M Fischbacher, B Guthrie, G Leese, R S Lindsay, J A McKnight, D Pearson, S Philip, N Sattar, S H Wild

**Affiliations:** 1 Centre for Population Health Sciences, University of EdinburghEdinburgh, UK; 2 Information Services Division, NHS National Services ScotlandEdinburgh, Aberdeen, UK; 3 Quality, Safety and Informatics Research Group, University of DundeeAberdeen, UK; 4 Diabetes and Endocrinology Department, Ninewells HospitalDundee, Aberdeen, UK; 5 British Heart Foundation Glasgow Cardiovascular Research Centre, University of GlasgowGlasgow, Aberdeen, UK; 6 Metabolic Unit, Western General HospitalEdinburgh, Aberdeen, UK; 7 Grampian Diabetes Research unit, University of AberdeenAberdeen, UK

## Abstract

**Aim:**

To describe characteristics associated with statin prescribing for the primary prevention of cardiovascular disease in people with newly diagnosed diabetes.

**Methods:**

Data from the Scottish Care Information—Diabetes Collaboration data set for 2006–2008 were used. This data set contains socio-demographic and prescribing data for over 99% of people with diagnosed diabetes in Scotland. Analyses were conducted on people aged over 40 years diagnosed with Type 1 or Type 2 diabetes between 2006 and 2008 with complete data and no previous history of cardiovascular or statin prescription. Logistic regression was used to calculate odds ratios for statin prescription in the 2 years following diagnosis of diabetes.

**Results:**

There were 7157 men and 5601 women who met the inclusion criteria, 68% of whom had a statin prescription recorded in the 2 years following diagnosis of diabetes. The proportions receiving statins were lower above 65 years of age in men and 75 years of age in women. People with Type 1 diabetes had lower odds of receiving statins than people with Type 2 diabetes [odds ratio (95% CI) 0.42 (0.29–0.61) for men and 0.48 (0.28–0.81) for women, after adjustment for age, BMI, smoking status, cholesterol level and deprivation]. Higher total cholesterol, BMI and being a current smoker were associated with greater odds of statin prescription.

**Conclusion:**

Approximately one third of the study population had no record of statin prescription during the 2 years after diagnosis of diabetes. Cardiovascular disease risk reduction opportunities may be missed in some of these people.

## What’s new?

1. This article reports the first use of Scottish Care Information – Diabetes Collaboration project data to examine which factors are associated with statin prescription in people during the first 2 years after diagnosis with diabetes in Scotland.
2. The results suggest that guidelines for the universal use of statins among people with diabetes were not being followed during the study period and that, as a result, opportunities to reduce cardiovascular disease risk were missed.
3. Decision support systems that prompt clinicians of risk reduction strategies could improve adherence to guidelines.

## Introduction

Diabetes mellitus is well established as a risk factor for cardiovascular disease and it is recommended that diabetes management should include attempts to prevent or delay the development of cardiovascular complications 1. Statins have been shown to reduce the risk of cardiovascular disease in people with diabetes and have become part of standard care 2. Diabetes and cardiovascular risk factors are more prevalent in older than younger age groups, in men than women and in deprived than affluent populations in developed countries and various risk scores take these factors into account 3,4. Inequitable use of effective treatments to reduce risk of cardiovascular disease could exacerbate inequalities in health.

The presence of clinical guidelines to aid decision making should support equitable care by recommending treatment based upon relevant clinical factors and cost-effectiveness. However the availability of guidelines will not necessarily result in equitable treatment 5. Despite this, guidelines can be used to identify a population of people who are recommended to receive treatment and investigate patterns of treatment and non-treatment within that group. Inequalities have previously been demonstrated in the management of coronary heart disease in the general population of Scotland 6], but it is not clear whether a similar pattern exists among people with diabetes in a contemporary population. In recent years, statins appear to be used more widely among deprived than affluent populations, but it is not clear whether statin use for primary prevention of cardiovascular disease is equitable among people with diabetes 7.

In 2005, the Joint British Societies (JBS) recommended that statins be offered to all people with diabetes to lower cholesterol, as the presence of diabetes alone was determined to identify high cardiovascular disease risk requiring appropriate management; in addition, the national guidelines from the Scottish Intercollegiate Guidance Network (SIGN) indicated that cardiovascular disease risk and consequent treatment ought to be based upon Joint British Society recommendations 8,9. Associations between socio-demographic factors and statin prescribing have been described in the general population of Scotland and in other settings 10,11 and socio-economic inequalities in cardiovascular disease mortality among people with diabetes persist in Scotland 12. The aim of this work was to investigate the patterns of statin prescribing in people with diabetes in Scotland, in order to establish what proportion of people with newly diagnosed diabetes receive statins for primary prevention of cardiovascular disease and whether there was equitable recording of statin prescribing.

## Methods

The Scottish Care Information – Diabetes Collaboration (SCI-DC) supports a national population-based electronic diabetes register containing clinical and demographic data for people with diabetes that is used to support clinical care and is updated daily 13. Individuals are included on this register if they are treated at either a Scottish hospital clinic or at one of approximately 99.5% of Scottish general practices, making the data set representative of people with diagnosed diabetes in Scotland. Approval for linkage of a pseudonymized Scottish Care Information – Diabetes Collaboration research data extract to other sources of health data including previous hospital admissions was obtained from the Scotland A Multi-centre research ethics committee, Caldicott guardians of all Health Boards and the Privacy Advisory Committee of NHS National Services Scotland. The data set used in this study is derived from a 2008 data extract that contains records for 272 074 people with diagnosed diabetes with an updated linkage of subsequent prescribing data in 2011.

### Variables used

Age, sex, BMI, smoking status, total serum cholesterol and type of diabetes (defined using an algorithm based on type recorded in clinical records, age at diagnosis of diabetes and treatment patterns) were used as covariates. BMI was divided into four categories: < 18.5, 18.5 to < 25, 25 to < 30 and ≥ 30 kg/m^2^, labelled as underweight, normal weight, overweight and obese, respectively. Age was divided into five categories: 40–54, 55–64, 65–74, 75–84 and ≥ 85 years. Total cholesterol was defined as high if it was ≥ 5 mmol/l. The area-based Scottish Index of Multiple Deprivation (SIMD) 2006 was used as a measure of socio-economic status, calculating an individual’s relative deprivation based upon income, employment, health, education, skills and training, housing, geographic access and crime in the postcode area of residence 14.

A binary dependent variable was developed from a file containing patients’ prescription records according to whether a person had a record of being prescribed a statin within the 2 years following diagnosis with diabetes.

### Statistical analysis

**Figure 1:**
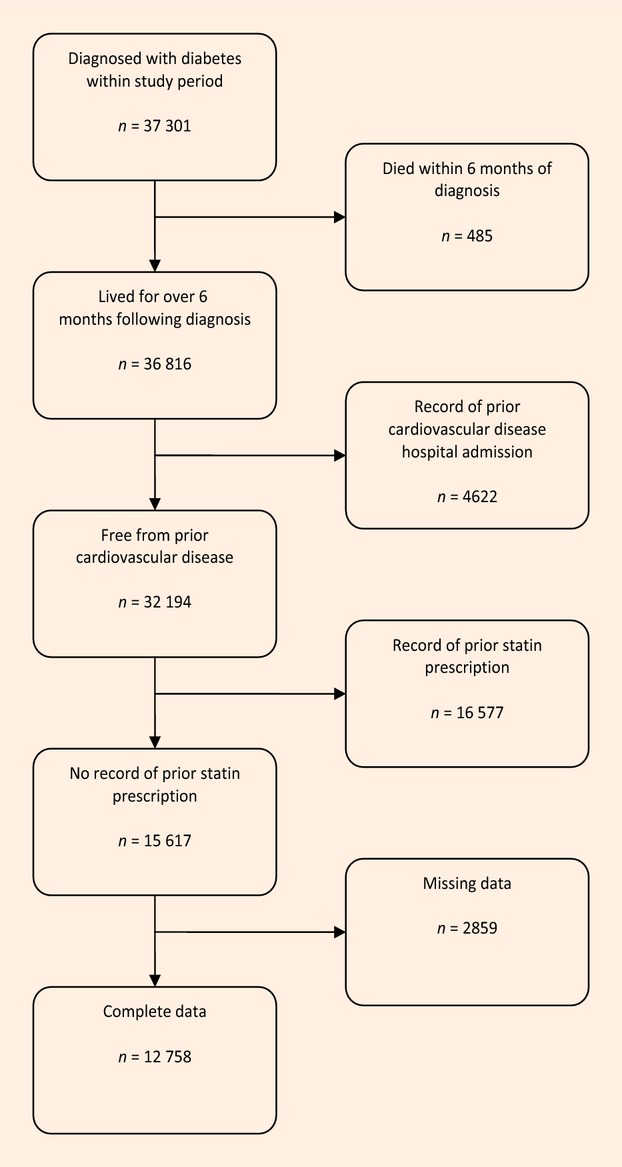
This data selection flow chart shows the process by which the study cohort were selected from the diabetes register.

The data used in the analysis were confined to those from individuals who were aged over 40 years and newly diagnosed with diabetes during the study period (1 January 2006 to 1 March 2008), with no recorded history of a hospital admission with cardiovascular disease or prior statin prescription, for whom complete data were available, and who did not die within 6 months of diagnosis (this selection process is illustrated in Fig.1). For measures that vary over time, for example total serum cholesterol, the measurement closest to the date of diabetes diagnosis was used. The follow-up period was for 2 years after diagnosis with diabetes.

We conducted sex-stratified logistic regression analysis on these data, with the reference categories as follows: deprivation = Scottish Index of Multiple Deprivation quintile one (the most deprived quintile); age = 40–54 years; diabetes type = Type 2 diabetes; cholesterol < 5 mmol/l; BMI = 18.5 to < 25 kg/m^2^; and smoking status = never smoked, as these were either the largest categories in their respective variables or were the most theoretically sound category to choose.

All analyses were conducted using Stata 11 (StataCorp., College Station, TX, USA).

## Results

There were 4622 people with a history of cardiovascular disease prior to diagnosis of diabetes within the study period, of whom 92% had a record of statin prescription, and 16 577 people who were already receiving a statin prescription for primary prevention of cardiovascular disease when a diagnosis of diabetes was made. People excluded from the analysis because they had missing data for one or more variables had a similar distribution of the other variables, although there was a lower proportion of current smokers (24% compared with 45%) among people whose data were excluded than those whose data were included. The data selection procedure is summarized in Fig.1.

There were 7157 men and 5601 women with incident diabetes between 1 January 2006 and 1 March 2008 who had no history of cardiovascular disease or previous statin prescription for whom complete data were available. Although all of these people were eligible for statin treatment based on contemporary guidelines, only 68% of men and 69% of women overall had a record of statin prescription in the 2 years following diagnosis with diabetes. The median time between diagnosis of diabetes and prescription of statins was 102 days.

The proportion of people prescribed statins varied by different characteristics (Table 1). There were higher proportions with a statin prescription record in people in the 55- to 64-years-of-age category than other age groups, in people with Type 2 compared with Type 1 diabetes, in people who were overweight or obese (BMI ≥ 25 kg/m^2^) compared with those who are normal or underweight (BMI < 25 kg/m^2^) and in current or former smokers than people who have never smoked, and in people with total cholesterol levels ≥ 5 mmol/l compared with < 5 mmol/l. The proportion receiving statins was highest in the most socio-economically deprived quintile.

**Table 1:** Proportion and number of people of over 39 years of age with no previous history of cardiovascular disease or statin prescription who had a statin prescription record within 2 years of diagnosis of diabetes in Scotland 2006–2008 by sex and population subgroup

	Men *n* = 7157 % (*n*)	Women *n* = 5601 % (*n*)
Age (years)		
40–54	70 (1989)	68 (1181)
55–64	71 (1610)	74 (1204)
65–74	65 (901)	72 (966)
75–84	55 (318)	61 (462)
≥ 85	33 (29)	31 (44)
Diabetes type		
Type 1	49 (63)	48 (30)
Type 2	68 (4768)	69 (3813)
BMI (kg/m^2^)		
< 18.5	49 (18)	40 (23)
18.5 to < 25	61 (528)	62 (444)
25 to < 30	68 (1728)	69 (1015)
> 30	69 (2573)	71 (2374)
Smoking status		
Never smoked	66 (1863)	67 (1919)
Current smoker	70 (1172)	74 (861)
Former smoker	69 (1812)	69 (1077)
Cholesterol (mmol/l)		
< 5	56 (1799)	56 (1149)
≥ 5	77 (3048)	76 (2708)
Scottish Index of Multiple Deprivation quintile		
1 (most deprived)	70 (1099)	73 (979)
2	69 (1058)	68 (887)
3	66 (945)	69 (796)
4	65 (896)	65 (627)
5 (least deprived)	69 (849)	67 (568)
Total	68 (4847)	69 (3857)

### Multivariate analysis

Table 2 shows the results of logistic regression analysis conducted separately for men and women, with the inclusion of age, type of diabetes, BMI category, smoking status, cholesterol level and deprivation quintile as covariates. Crude and age-adjusted odds were very similar and, as a consequence, the relationships found between statin prescription and the covariates are very similar to those reported above. Higher odds of a record of a statin prescription were found for people in the 55- to 64-years-of-age category than other age groups, in people who were overweight or obese (BMI ≥ 25 kg/m^2^) compared with those who are normal or underweight (BMI < 25 kg/m^2^) and in current or former smokers than people who have never smoked, with the highest odds ratio noted for people with total cholesterol levels ≥ 5 mmol/l compared with < 5 mmol/l. Lower odds of a record of a statin prescription were found for people with Type 1 diabetes compared with people with Type 2 diabetes, but small numbers of people with incident Type 1 diabetes over 40 years of age result in wide confidence intervals. There was no clear pattern of statin prescribing with socio-economic status, although women in the most deprived quintile had higher odds of having a statin prescription recorded than women in other quintiles.

**Table 2:** Odds ratios for statin prescription in the study population derived from a multivariate model containing age, diabetes type, BMI, smoking status, cholesterol level and deprivation quintile for men and women

	Men *n* = 7157 Nagelkerke’s *r*^2^ = 0.096				Women *n* = 5601 Nagelkerke’s *r*^2^ = 0.101			
	Odds ratio	*P*-value	Confidence interval		Odds ratio	*P*-value	Confidence interval	
			Lower	Upper			Lower	Upper
Age (years)								
40–54	1.00				1.00			
55–64	1.11	0.115	0.98	1.26	1.35‡	0.000	1.15	1.58
65–74	0.87	0.063	0.75	1.01	1.29†	0.002	1.10	1.52
75–84	0.58‡	0.000	0.48	0.71	0.80*	0.027	0.66	0.98
≥ 85	0.25‡	0.000	0.15	0.40	0.24‡	0.000	0.16	0.36
Diabetes type								
Type 1	0.42‡	0.000	0.29	0.61	0.48†	0.006	0.28	0.81
Type 2	1.00				1.00			
BMI (kg/m^2^)								
< 18.5	0.52	0.064	0.26	1.04	0.44†	0.005	0.24	0.78
18.5 to < 25	1.00				1.00			
25 to < 30	1.23*	0.019	1.03	1.45	1.26*	0.025	1.03	1.53
≥ 30	1.21*	0.026	1.02	1.43	1.19	0.061	0.99	1.44
Smoking status								
Never smoked	1.00				1.00			
Former smoker	1.21†	0.002	1.07	1.36	1.05	0.526	0.91	1.20
Current smoker	1.15	0.050	1.00	1.32	1.25†	0.007	1.06	1.47
Total cholesterol (mmol/l)								
< 5	1.00				1.00			
≥ 5	2.60‡	0.000	2.34	2.89	2.47‡	0.000	2.19	2.78
Scottish Index of Multiple Deprivation quintile								
1 (most deprived)	1.00				1.00			
2	1.03	0.738	0.88	1.20	0.78†	0.006	0.66	0.93
3	0.85	0.054	0.73	1.00	0.83*	0.040	0.69	0.99
4	0.85*	0.045	0.72	1.00	0.68‡	0.000	0.56	0.82
5 (least deprived)	0.99	0.931	0.84	1.18	0.73†	0.002	0.60	0.89

*P < 0.05, ^†^*P* < 0.01, ^‡^*P* < 0.001.

## Discussion

Although contemporary guidelines recommended that all people with diabetes should receive statin treatment 8, we found that 68% of people diagnosed with diabetes between January 2006 and March 2008, without a record of prior cardiovascular disease or statin prescription, were prescribed a statin for primary prevention of cardiovascular disease within the 2 years following diagnosis of diabetes. Characteristics associated with higher risk of cardiovascular disease other than age and type of diabetes, including low socio-economic status, a high BMI, current smoking status and high total cholesterol, were associated with increased odds of a record of statin prescribing. The strongest association was between statin prescription and having a total cholesterol level equal to or in excess of 5 mmol/l, which may partly be an indication of the effectiveness of the Quality of Outcomes Framework (QOF), because the Quality of Outcomes Framework provides incentives in primary care for prescribing statins to people with a cholesterol measurement in this range. However, it should be noted that SIGN guidance recommends statin prescription in Type 2 diabetes > 40 years irrespective of cholesterol level 9.

The finding that the odds of treatment were greater in men and women with a BMI ≥ 25 kg/m^2^, male former smokers, female current smokers, people with total cholesterol ≥ 5 mmol/l and people from more deprived areas compared with relevant reference groups indicates that people at higher risk of cardiovascular disease within the study population are more likely to receive a statin prescription than those at lower risk.

Despite the small numbers involved, the odds of having a statin prescription recorded for people with Type 1 diabetes were statistically significantly lower than for people with Type 2 diabetes. SIGN guidelines covering this period suggest treatment at a lower risk threshold for patients with Type 1 diabetes, because of the potential for underestimation in the current risk assessment methods for these patients. However, current guidelines state that statins for primary prevention of cardiovascular disease ‘are recommended’ for people with Type 2 diabetes and ‘should be considered’ for people with Type 1 diabetes. If this is not a chance finding, it may reflect lack of evidence of effectiveness of statins, or uncertainty about risk of cardiovascular disease among people with Type 1 diabetes.

There is evidence from several trials, which contained both people with and without diabetes, that statin treatment can be beneficial up to 80 years of age 15–17. Despite this, we found that men above 65 years of age and women above 75 years of age had lower odds of receiving statins than younger people, a finding which is consistent with those of other studies 18–20. This could be attributable to a variety of reasons, including co-morbidity, poly-pharmacy, concerns about side effects or effectiveness of statin treatment in older people, given that the time taken for the benefits of statin treatment to accrue could be greater than an individual’s life expectancy, and recognition of lower relative risks of mortality associated with diabetes that develops at older compared with younger ages. This finding contrasts with the overall pattern observed in these results, because people at greater absolute cardiovascular disease risk attributable to higher age had lower odds of a record of statin prescribing, whereas people with a high cardiovascular disease risk indicated by the other factors had greater odds of having a statin prescribing record.

Our finding that there was a non-linear pattern of statin prescription by socio-economic status is not consistent with the inverse care law that describes poorer treatment for the people at highest risk 21. The finding of greater proportions of more deprived people receiving treatment with statins is consistent with a study reporting treatment patterns for both primary and secondary prevention of cardiovascular disease among a general population 7.

Although this study has investigated potential undertreatment with statins, it is worth noting that a recent study has found that some people with diabetes may be overtreated with statins, receiving an excessive dosage 22. In future, this work could be extended to investigate statin doses and whether this issue is also present in this population.

### Comparison with previous studies

A study that used data from across Scotland to examine the management of coronary heart disease found that, between 1998 and 2001, the most deprived patients (also defined by quintiles of Scottish Index of Multiple Deprivation) had lower odds of receiving a statin than the least deprived (odds ratio 0.7, 95% CI 0.4–1.2) 6. The population and time period differ, but it is interesting that this study found a differing pattern. This may be explained by increasing awareness of deprivation as a risk factor 23 and the introduction of the Quality of Outcomes Framework, one of the aims of which was to reduce health inequalities 24. These have been associated with a substantial increase in the quantity of lipid-lowering medication prescribed in Scotland between 2003 and 2007 25.

An analysis of statin prescribing in England found that general practices in more deprived areas were prescribing statins to a larger proportion of their patients than were practices in less deprived locations, even after adjustment had been made for differences in disease prevalence between these areas, which corresponds with the social gradient reported here 7. However, there is great potential for these results to be attributable to an ecological fallacy because this analysis was conducted at a practice rather than individual level.

A 2009 study from Norway discovered that, following adjustment for age, sex and cardiovascular disease risk factors, people in the highest education category (a proxy for socio-economic status) were more likely to start using statins than people in the lowest education category (relative risk 1.35, 95% CI 1.00–1.81) 26, whilst an investigation of statin prescription in Italy found that people with an intermediate education level (of three levels) had higher prescription rates than people with the lowest education level (prevalence rate ratio** **1.11, 95% CI 1.04–1.19) 27. Whilst educational attainment is not strictly comparable with the more complex index of deprivation used in our study, it is a common measure of socio-economic status, and these findings indicate that, in these instances, a lower socio-economic status was associated with less aggressive treatment, which is the opposite of the socio-economic relationship we found and is indicative of a more typical form of inequity. Similar results were found in Denmark in a 2005 paper where men of a higher socio-economic status were found to have a greater likelihood of receiving statins than men of a lower socio-economic status (relative prevalence proportion 1.85, 95% CI 1.17–2.96), whilst the likelihood for women was not affected by socio-economic status 10.

In addition to the differences in population, research question and study design, the findings of research conducted in other countries are likely to be markedly different given differences in healthcare systems. Despite this, it is still of interest to note the variability in statin prescription and that its relationship with socio-economic status is not consistent in its direction over time or between different places. It is also interesting to note that statins are not universally used, even in clinical trials among people with diabetes and cardiovascular disease (for example, only approximately 75% of patients in the coronary artery bypass graft arm and 87% of people in the percutaneous coronary intervention were prescribed statins after the study procedure in the SYNTAX trial among patients with three-vessel or left main coronary artery disease, and only 86–95% of patients were on statins in the COURAGE trial, which compared optimized medical therapy with percutaneous revascularization in patients with stable coronary artery disease 28,29).

### Study strengths

This study draws its data from a population-based data set containing records for nearly all people with diagnosed diabetes in Scotland. It is the first to have investigated factors associated with statin prescribing in relation to contemporary guidelines among people with diabetes in Scotland. The outcome of interest was ever having received a prescription after diagnosis of diabetes, so that people who were unable to tolerate a statin but who ever received a prescription were included. The limited use of lipid-lowering treatments other than statins among people with diabetes in Scotland (approximately 1% of the population with diabetes have a record of one or more such prescriptions) means that use of these treatments is unlikely to confound our findings.

### Study limitations

A limitation of this study was the lack of information about non-pharmaceutical attempts to lower risk that may have been offered, which means that we could not ascertain whether patients with a lower perceived risk were being instructed to modify their lifestyle instead of being treated with statins, rather than simply receiving no treatment (although it should be noted that this would still not be suitable treatment according to the Joint British Society or SIGN guidelines). In addition, we only considered a limited number of cardiovascular disease risk factors and did not investigate records of statin prescription in strata of ethnicity, blood pressure, total:HDL cholesterol, albuminuria/proteinuria and HbA_1c_ values that may also influence statin prescribing. This study was limited by not being able to investigate the relationship between clinician, practice or hospital characteristics and the decision not to prescribe. We also do not have information about patients who decline to receive a statin prescription offered by their clinician, which might affect different population groups, perhaps younger people, to a greater extent. The lack of data concerning over-the-counter statin purchases may also be a problem and have a differential effect by socio-economic status, but, given that prescribed statins were free for the majority of this population (people with diabetes treated with medication or insulin were exempt from all prescription charges in the study period), this is unlikely to have had a large effect. This study is also limited by variation between different sets of clinical guidelines, which means that it may not be appropriate to apply a single standard.

## Conclusions

Despite contemporary recommendations that all people with diabetes should be treated with statins, only 68% of people with incident diabetes in Scotland who had not been taking statins before diagnosis of diabetes had a record of a first statin prescription for primary prevention of cardiovascular disease within 2 years of follow-up. The odds of statin prescribing varied with patient characteristics, such that people at higher risk of cardiovascular disease from factors other than age were more likely to receive statins than those at lower risk, suggesting that clinical judgement is used in applying guidelines. However, it is possible that an opportunity for cardiovascular disease risk reduction using statin therapy is being missed in some people with diabetes. Finally, we found no evidence that the pattern of prescribing was systematically disadvantaging people from more socio-economically deprived populations.

### Funding sources

This work was funded by the Wellcome Trust through the Scottish Health Informatics Programme (SHIP) Grant (grant no. WT086113). Funding for diabetes register linkage was provided by the Scottish Government and the authors acknowledge the financial support of NHS Research Scotland (NRS), through the Scottish Diabetes Research Network.

### Competing interests

RSL has received advisory board payments from Novo-Nordisk and Lilly. NS has served on Advisory boards for BMS, Astranzeneca and MSD in relation to lipid-lowering therapy and received grant funding from Pfizer.
